# Supplementary material for: Prognostic Impact of Heat Shock Protein 90 Expression in Women Diagnosed with Cervical Cancer
Source: Int J Mol Sci. 2024 Jan 26;25(3):1571. doi: 10.3390/ijms25031571 (PMC10855426; doi:10.3390/ijms25031571)
Supplement: Supplementary file 1 [file ijms-25-01571-s001.zip › ijms-2825515-supplementary.pdf]

Table S1. Correlation of HSP90 expression and various histopathological variables. (* $p < 0.05$, ** $p < 0.001$)

	HSP90 (nucleus)		HSP90 (cytoplasm)	
	**Correlation coefficient**	***p***	**Correlation coefficient**	***p***
HSP90 (nucleus)	-	-	0.422	**< 0.001** **
HSP90 (cytoplasm)	0.422	**< 0.001** **	-	-
RIPK1 (nucleus)	0.309	**< 0.001** **	0.228	**0.001** *
RIPK1 (cytoplasm)	0.410	**< 0.001** **	0.426	**< 0.001** **
RIPK3 (nucleus)	0.517	**< 0.001** **	0.422	**< 0.001** **
pMLKL (nucleus)	0.135	0.058	0.118	0.093
pMLKL (cytoplasm)	0.137	0.054	0.115	0.101
E6 (cytoplasm)	*-0.059*	0.378	0.138	**0.042** *
p16 (cytoplasm)	0.093	0.174	*-0.081*	0.237
p53 (nucleus)	0.208	**0.002** *	0.254	**< 0.001** **
p53 (cytoplasm)	*-0.040*	0.558	*-0.150*	**0.025** *
Mutated p53 (nucleus)	0.178	**0.007** *	0.176	**0.008** *
Mutated p53 (cytoplasm)	*-0.042*	0.530	*-0.205*	**0.002** *
p21	0.229	**0.002** *	0.180	**0.015** *
